# Adult Hippocampal Neurogenesis in Alzheimer's Disease: An Overview of Human and Animal Studies with Implications for Therapeutic Perspectives Aimed at Memory Recovery

**DOI:** 10.1155/2022/9959044

**Published:** 2022-01-15

**Authors:** Stefano Farioli-Vecchioli, Valentina Ricci, Silvia Middei

**Affiliations:** ^1^Institute of Biochemistry and Cell Biology, National Research Council, Via E. Ramarini 32, Monterotondo, 00015 Rome, Italy; ^2^European Brain Research Institute, Via Regina Elena 295, 00161 Rome, Italy

## Abstract

The mammalian hippocampal dentate gyrus is a niche for adult neurogenesis from neural stem cells. Newborn neurons integrate into existing neuronal networks, where they play a key role in hippocampal functions, including learning and memory. In the ageing brain, neurogenic capability progressively declines while in parallel increases the risk for developing Alzheimer's disease (AD), the main neurodegenerative disorder associated with memory loss. Numerous studies have investigated whether impaired adult neurogenesis contributes to memory decline in AD. Here, we review the literature on adult hippocampal neurogenesis (AHN) and AD by focusing on both human and mouse model studies. First, we describe key steps of AHN, report recent evidence of this phenomenon in humans, and describe the specific contribution of newborn neurons to memory, as evinced by animal studies. Next, we review articles investigating AHN in AD patients and critically examine the discrepancies among different studies over the last two decades. Also, we summarize researches investigating AHN in AD mouse models, and from these studies, we extrapolate the contribution of molecular factors linking AD-related changes to impaired neurogenesis. Lastly, we examine animal studies that link impaired neurogenesis to specific memory dysfunctions in AD and review treatments that have the potential to rescue memory capacities in AD by stimulating AHN.

## 1. Introduction

Human adult neurogenesis, the generation of new neurons from neural stem cells in specific areas of the adult brain, has been at the center of an intense scientific research over the past years. New neurons are continuously generated in the human hippocampal dentate gyrus (DG), a brain region involved in learning and memory. This ongoing generation peaks at a young age but declines in adulthood and drops in old age, when memory decline also commonly occurs. The temporal correlation between reduced adult hippocampal neurogenesis (AHN) and impaired memory has been the rationale for animal studies investigating whether and how hippocampal newborn neurons contribute to memory. The general view that emerged from these studies is that new neurons are involved in distinct mechanisms of memory [1, 2].

The impact of ageing on altered AHN and the associated cognitive decline has encouraged researchers to investigate the possibility that deficits in AHN are a complicating factor in Alzheimer's disease (AD), the most frequent form of dementia and memory loss in ageing individuals. This possibility has been intensively debated, due to the lack of a clear and homogeneous methodology for the identification of new neurons in human hippocampal tissues. However, recent studies [3–5] provided convincing evidence for a massive decay in AHN in AD brains and shifted the focus of the debate towards new scientific questions concerning therapeutic approaches that can reinforce neurogenesis in AD patients.

Thus, the present paper is aimed at providing a state-of-the-art review on studies that link AHN to memory in AD, as well as at delineating the questions that in our opinion should be addressed by scientific and clinical research in the near future. We review both relevant experimental investigations about adult neurogenesis in AD patients from the last two decades and studies on mouse models of AD. Although direct comparisons between humans and rodents cannot be made because of the huge species-specific variability [6], we will extrapolate from animal studies key information to understand neurogenesis in AD patients. We then focus on factors and therapeutic approaches that have the potential to trigger neurogenesis to contrast AD. Given the relevance of AHN for memory, we will limit our discussion to the hippocampus only.

## 2. Neurogenesis

### 2.1. Key Steps of Adult Hippocampal Neurogenesis

**Figure 1:**
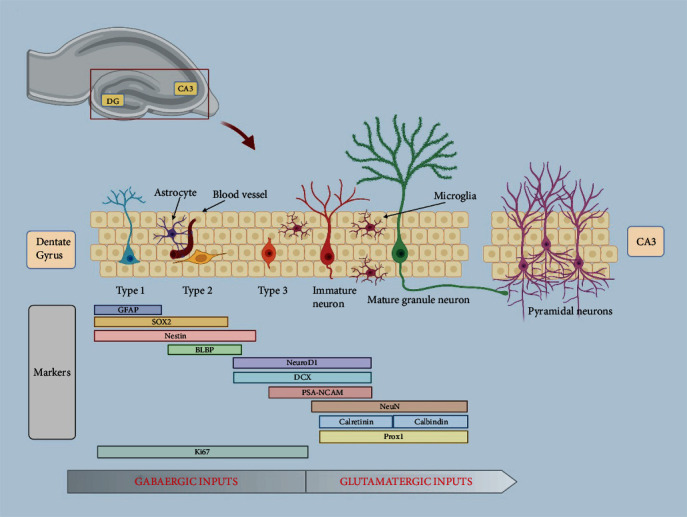
Main steps of AHN. Top left: image representing the position of the dentate gyrus (DG) and its main output target, the CA3 subregion, within the hippocampal circuit. Middle panel: morphological characterization and main markers related to the different cell types that identify the transition of a newborn neuron from a neural stem cell to a mature neuron integrated into a pre-existing circuit. As indicated by the scheme, the different stages undertaken by a newly generated neuron within the hippocampal dentate gyrus are characterized by a peculiar morphological identity and by the expression of specific cell markers (see boxes) that make it possible to study the proliferative and differentiative dynamics finely orchestrating the maturation of newborn neuron. Bottom diagram: neurotransmitters involved in the differentiation and maturation processes of newborn neurons. A first phase characterized by an excitatory GABAergic signaling that enables the maturation of the neural progenitors is followed by an excitatory glutamatergic signaling, which will permanently distinguish the electrophysiological properties of the new-generated granule neurons in the hippocampal dentate gyrus circuit. GFAP: glial fibrillary acidic protein; Sox2: SRY- (sex determining region Y-) box; BLBP: brain lipid binding protein; Neuro D1: neuronal differentiation 1; DCX: doublecortin; PSA-NCAM: polysialylated-neural cell adhesion molecule; NeuN: neuronal nuclear protein; Prox 1: prospero homeobox 1; Ki67: proliferative marker.

Since the discovery of adult neurogenesis, intensive research in rodent studies has been investigating the steps through which quiescent adult neural stem cells (qNSCs) become new mature neurons functionally integrated in the hippocampal trisynaptic circuit (Figure 1). Radial glia-like (RGL) NSCs (also known as type 1 cells) are located in the subgranular zone (SGZ), a restricted region of the hippocampal dentate gyrus (DG) on the border between the granule cell layer (GCL) and the hilus. This narrow area ensures an essential environmental niche where complex signaling pathways and support cells (astrocytes, microglia, and endothelial cells) allow the RGLs to maintain their quiescent state (qNSCs). Following appropriate intrinsic and/or extrinsic stimuli, the neurogenic niche plays a fundamental role in modulating the recruitment of qNSCs in the cell cycle, and in promoting the necessary differentiation/maturation steps and overseeing the functional integration of newly generated neurons.

The use of markers expressed specifically in the different subpopulations originating from the qNSCs and neural progenitors allows the defining of the cell lineage in hippocampal neurogenesis. Through this approach, studies have established that type 1 NSCs (expressing the specific markers GFAP, Nestin, SOX2, and BLBP) give rise to type-2 amplifying progenitors, which manifest their neural commitment, as evidenced by the coexisting expression of transcription factors NeuroD1 and Prox-1, as well as of the structural protein Doublecortin (DCX).

Proliferative type-2 cells differentiate into type-3 neuroblasts (characterized by the expression of DCX, NeuroD1, and PSA-NCAM), which exit from the cell cycle and start their migration towards the inner layer of the GCL, where they mature into granule cells by extending long axonal projections along the mossy fiber path. In the final maturation phase, the newborn neurons, which are specifically recognized by the expression of NeuN, Calbindin, and Prox1, send their axonal projections toward the CA3 layer of pyramidal neurons, providing an essential cue for the integration in the hippocampal circuitry [7, 8].

Under physiological conditions, adult hippocampal neurogenesis generates only one type of neuron, the granule cells, which represent the main glutamatergic excitatory neurons of the DG. Recent studies demonstrate that progenitor cells initially receive excitatory GABAergic synaptic inputs, which facilitate their maturation. About three weeks after birth, the response to GABA changes from depolarization to hyperpolarization, which corresponds to the beginning of glutamatergic excitatory signaling [9, 10].

### 2.2. Evidence of Adult Hippocampal Neurogenesis in Humans

The presence of newly generated neurons in the human hippocampus has raised widely contrasting, sometimes antithetic, results from different research groups. The first report of human hippocampal neurogenesis has been documented in 1998 by Eriksson et al. In this study, BrdU (a dye that intercalates DNA during cell division) was administered to 5 terminal cancer patients and 1 subject control, and the presence of positive BrdU cells (BrdU^+^) was found in the postmortem autoptic DG samples [11]. However, the small sample size and the unethical implications of the study raised both doubts and contradictions concerning these results. A couple of decades later, Spalding et al. [12] employed carbon-14 dating to estimate the age of neurons in postmortem tissues of 55 people aged 15-92. Based on this indirect procedure, the study assessed the production of about 700 new neurons every day in the hippocampus of middle-aged men. It was then suggested that about 35% of the hippocampal cells renew during a lifespan, with an estimated turnover of around 2% every year [12, 13].

Over the last few years, three relevant publications reopened the debate about adult hippocampal neurogenesis. One of these studies [14], based on immunofluorescence examination of autoptic hippocampal samples, stated that there was no evidence of hippocampal neurogenesis from an adolescent stage onward. This “denial” study has raised a number of animated methodological criticisms concerning tissue selection and preservation, including (a) the scarcity of information about perimortem causes, which might affect tissue preservation; (b) the 48 hours postmortem delay (PMD), i.e., the time elapsed between death and brain fixation, which could be associated with protein rupture and the consequent disappearance of the antigenicity of several markers including DCX [15] the main neurogenic marker used in human studies; and (c) the morphometric analysis, which was run on a small number of samples randomly chosen and not taking into consideration that neurogenesis differs considerably between the dorsal and ventral region of the hippocampus.

On the ground of these and other remarks, many researchers rejected the idea that hippocampal neurogenesis in humans is interrupted during adolescence, as previously observed in the subventricular zone [6].

Two other studies [3, 16] then clearly demonstrated that adult neurogenesis in the human hippocampus is a robust process, which ensures a continuous supply of new neurons during adult life. However, while Boldrini et al. [16] showed that neurogenesis persists at high levels in the elderly, the work by Moreno-Jimenez et al. [3] stated that the generation of new neurons declines with age, with a greater extent in the brains of Alzheimer's patients. The latter study reported the coexistence of numerous neural progenitor-associated markers (including DCX, PS-NCAM, and Prox-1) in DG single cells of 13 healthy individuals and received an almost unanimous scientific recognition for the optimization of some steps in the utilized protocol. For instance, brain samples were selected after a very short PMD and were maintained in fixation for a long time. Furthermore, autofluorescent quenching was significantly reduced while epitope retrieval and antibody selection were optimized. These methodological improvements allowed the authors to observe a higher number of DCX-positive (DCX^+^) progenitors in the middle-aged DG as compared to similar studies [3].

Finally, Tobin et al. [5] confirmed the existence of hippocampal neurogenesis in 18 postmortem brains of elderly people ranging from 79 to 99 years of age. The relevance of this study lies in establishing with extreme clarity the presence of proliferation (PCNA^+^ and Ki67^+^ cells) both in NSCs (Nestin^+^ and SOX^+^ cells) and in DCX^+^ neural progenitors. Furthermore, this study pointed to a regional cell distribution with proliferating and progenitor cells localized along the dorsal-ventral axis, while the NSCs (Nestin^+^) were distributed preferentially in the dorsal portions of the hippocampus. In conclusion, at the time of writing this review, a fairly supported agreement has been reached about the persistence of adult neurogenesis in the human brain, although with some discrepancies concerning the decline rate occurring with ageing.

### 2.3. The Contribution of Newborn Neurons to Memory

Computational models hypothesized the involvement of AHN in three key memory processes known as pattern integration, pattern separation, and memory erasure. Here, we describe these phenomena and provide experimental data from behavioral studies in rodents.

Associative memories are generated by the co-occurrence of two or more events within a limited time window. The neurobiological mechanism sustaining this process, known as pattern integration, consists in the simultaneous activation of distinct DG cells in response to coincident events. Given their peculiar hyperexcitability, newborn neurons are extremely efficient in detecting temporally related events [17, 18], which makes these cells pivotal for pattern integration. Indeed, reduction of DG neurogenesis has been shown to compromise mice's ability to pair both object-place associations in the object location task [19] and auditory (or visual) stimulus with an unconditioned stimulus in the eyeblink conditioning task [20]. Furthermore, loss of DG neurogenesis leads to low performance in contextual fear conditioning, a task in which rodents learn to associate an aversive event with the context in which it takes place [21, 22]. During memory encoding, the disambiguation of similar contexts through a process known as pattern separation allows the formation of a precise and accurate memory. Pattern separation depends on sparse activity of DG neurons leading to low network activity, which is implemented by highly excitable newborn neurons [23, 24]. In fact, reducing neurogenesis decreases overall DG inhibition while increasing it leads to interneurons activation and bigger DG inhibition [25, 26]. Hence, highly excitable newborn inhibitory neurons may act to modulate mature DG cells leading to a sparse activation necessary for pattern separation.

Consistently, deletion of newborn neurons in rodents compromises their pattern separation ability. Studies have shown that ablating AHN in mice resulted in impaired radial arm maze performance if surrounding contextual cues were presented in a complex spatial configuration with little spatial separation [27] and in reduced discrimination between two distinct contexts in fear conditioning [28]. Suppression of AHN also impaired performance in the Morris water maze (MWM), a task in which mice must learn the position of a submerged platform in order to escape from the water. As a further support to the idea that newborn neurons are necessary for pattern separation, increased discrimination between similar contexts was reported after enhancing AHN through genetic tools [29] or voluntary running [30].

Proactive interference is the process by which one encoded information overlaps with a new one limiting the possibility for both new memory encoding and old memory retrieval. The above-mentioned process of pattern separation acts to reduce proactive interference, but neurogenesis-associated clearance of previously existing memories can also play a role. In fact, one relevant study in rodents has demonstrated that enhancing AHN by wheel running reduces old contextual fear memory [31]. Avoiding the interference between old and new memories is also necessary for cognitive flexibility, which is the ability to adapt a new behavioral strategy to face environmental changes. This form of reversal learning, which has been examined in the MWM by moving the platform from a familiar to a novel position, was facilitated in mice with enhanced AHN [32].

## 3. Neurogenesis in AD

### 3.1. Evidence of Altered Neurogenesis in AD Patients

Alzheimer's disease (AD) is the most common neurodegenerative disease associated with ageing. The hippocampus and entorhinal cortex, two key regions for memory, are particularly vulnerable to AD neurodegeneration. Clinically, AD patients manifest a severe impairment in cognition that principally affects memory functions. Several studies have examined whether altered AHN plays a role in AD progression. However, contrasting results were obtained, likely due to both the scarcity and heterogeneity of the brain samples analyzed, and to the difficulty of preservation and immune-detection analysis of the tissues of postmortem brains.

Some studies in the first decade of the 2000s reported intact adult neurogenesis in patients with presenile AD [33], or even a sharp increase of proliferation in neuroblasts expressing the specific markers DCX, PS-NCAM, and Tuc-4 [34].

However, a reduced hippocampal neurogenesis was observed in most studies in AD patients [3, 5, 35–38]. In particular, Crews et al. [37] reported a sharp decrease in hippocampal neurogenesis, which was closely related to a significant increase in the expression levels of the growth factor BMP6, especially in proximity of the A*β* plaques [37]. Another study found that adult neurogenesis abnormalities in AD patients vary considerably between neurogenic stages and disease progression with a net decrease of stem cell number compensated by an enhanced proliferation. However, this enhanced proliferation did not lead to an increased number of new differentiated neurons [38]. More recently, two papers have provided additional information about this topic, comparing a fairly large number of brain samples of elderly individuals and AD patients. In both studies, what emerged quite unequivocally was a sharp drop of hippocampal neurogenesis in AD patients with respect to control individuals [3, 5]. In detail, Tobin et al. [5] observed a reduced number of neuroblasts at early stages of cognitive decline. Interestingly, these authors revealed a direct correlation between the number of proliferating neuroblasts (DCX^+^, PCNA^+^) and clinical diagnosis, observing a significantly reduced number of neuroblasts in the early stage of mild cognitive impairment (MCI). Based on this evidence, the authors speculated that depletion of hippocampal neurogenesis might represent an early neuropathological aspect that promotes or exacerbates cognitive deficits in AD [5].

Hence, a correlation between decreased adult neurogenesis and AD seems now established with a good degree of certainty, although it remains to be ascertained whether the neurogenic decline has a causal role or is a consequence of the AD-dependent neurodegenerative events.

### 3.2. Mouse Models to Understand the Mechanisms of Impaired Neurogenesis in AD

The hallmarks of AD brains include plaques made of fragments derived from amyloid precursor protein (APP) and intracellular aggregates of hyperphosphorylated TAU protein, respectively, known as amyloid plaques and neurofibrillary tangles. Most of the altered genes in the familiar forms of AD (FAD) relate to the expression of proteins involved in APP cleavage or clearance of its proteolysis products. Below, we review studies linking pathological neurogenesis processes to the expression of APP or to FAD-related genes involved in altered APP proteolysis. Furthermore, we discuss the relationship between AHN and hyperphosphorylated TAU or apolipoprotein E (ApoE), a key risk factor for AD.

#### 3.2.1. Amyloid Precursor Protein (APP)

APP is a membrane-bound protein involved in several physiological functions. Its role in neurogenesis has been assessed in studies exploring the effects of either overexpression or deletion of wild type APP in rodents.

In mice overexpressing APP, the number of BrdU-labeled (BrdU^+^) cells was reduced as compared to controls but it increased after exposure to environmental enrichment, a condition that strongly potentiates cells differentiation [39]. On the contrary, mice with APP deletion displayed more BrdU^+^/DCX^+^ cells relative to controls, suggesting that APP restricts NPG cell proliferation. This modulation is likely acting through GABA regulation of stem cell quiescence [40] since selective deletion of APP from GABAergic neurons was sufficient to increase progenitor proliferation [41]. The number of proliferating cells that differentiate into neurons (BrdU^+^/NeuN^+^ cells) was comparable between mice overexpressing APP and wild-type ones, but in the former genotype, this number was reduced with ageing [41, 42]. Furthermore, dendritic length and branching were found reduced in differentiated neurons of APP KO mice [41, 42]. Overall, this set of evidence indicates that APP exerts a control function on proliferation, differentiation, and maturation of newborn cells.

#### 3.2.2. APP Proteolytic Products

Age and genetic factors, including Presenilin1 (PS1) mutations, are at the origin of APP processing into proteolytic products that gradually accumulate in amyloid plaques. One study directly addressing whether the presence of amyloid plaques alters hippocampal neurogenesis was conducted in the plaque-bearing Tg2576 mouse model. This study reported that a reduced number of BrdU^+^ cells was already evident in the DG of mutant mice before plaque appearance, while pharmacological rescue of neurogenesis did not alter plaque load [43]. This evidence, which indicates that the production of new neurons is already impaired before plaque manifestation, has been confirmed by other studies on mouse models of AD-like progression (see Table 1). In apparent contrast, one study using the APP/PS1ΔΕ9 mouse model [44] found that treating mutant mice with metformin resulted in both reduced plaque load and enhanced neurogenesis. However, it is likely that these events are unrelated and are linked to a common third factor, i.e., the inhibition of reactive astrogliosis and microgliosis induced by metformin.

Plaques are mainly composed of A*β*, a product of APP proteolysis that gradually aggregates into oligomers and fibrils. A*β* has been considered as potentially harmful for neurogenesis in AD by several studies documenting impaired neurogenesis in transgenic mice expressing the PS1 mutation, which dramatically increases A*β* levels long before plaque appearance (Table 1). More direct evidence of this hypothesis is that A*β*_1-42_ delivery in brain ventricles of wild type mice resulted in reduced BrdU^+^/NeuN^+^ cell numbers as compared to controls [45]. Furthermore, treatments that drop A*β* levels, including *β*2-adrenergic agonist [46] or a drug that blocks amyloidogenic APP cleavage [47], are effective in restoring neurogenesis in AD mice.

Although the above evidence implies that the presence of A*β* interferes with neurogenesis, other studies lead to opposite conclusions. Wang et al. [48] reported a reduced number of BrdU^+^ DG cells in 3xTgAD mice as compared to controls at 3 months of age, which is before the occurrence of A*β* signs in these mice. One other study [49] compared neurogenesis between hAPP-J20 and hAPP-I5 mice, which display different A*β* amounts. Although A*β* levels were higher in hAPP-J20 mice, survival of newborn cells was more compromised in hAPP-I5. The authors provided further support to the idea that impaired neurogenesis was not associated with A*β* levels by demonstrating that deletion of cystatin C, which is known to reduce A*β*, did not alter the number of DCX^+^ neurons in hAPP-J20 mice.

Valero et al. [50] exposed A*β* overexpressing mice to the highly neurogenic condition of an enriched environment. Although A*β* levels remained unvaried upon enriched environment, this condition increased the number and complexity of newborn neuron projections to CA3, meaning that newborn neurons were integrated in the hippocampal circuit.

Several other studies have investigated neurogenesis in mice expressing mutations that contribute to amyloidogenic processing of APP (Table 1), leading to mixed results. The disparity among these correlative studies is likely due to differences in transgene expression, mouse line, animal age, or other experimental conditions. Despite discrepancies, some of these studies are noteworthy as they highlight several mechanisms that are triggered by A*β* and may affect neurogenesis. Among these, A*β* alters the expression neurotrophins BDNF, NGF, and NT3, as well as the expression and phosphorylation of receptors TrkA and TrkB in mice with reduced neurogenesis [51, 52]. Given the importance of neurotrophins in neurogenesis [53, 54], this evidence suggests that A*β* may act as a toxic agent that interferes with neurotrophic mechanisms. In fact, AHN reduction in AD mice could be rescued by treatment with L-NPB [52] or Osthole [51], two molecules that increased the levels of neurotrophins involved in neurogenesis. One other study [55] confirmed these results in APP751 mice, by showing that administration of Cerebrolysin, a peptidergic mixture that increases BDNF levels, improved survival of grafted NSCs in the DG. One possible mechanism involved in impaired neurogenesis lies in the amyloid-associated microgliosis and the induction of proinflammatory cytokines as documented in studies reporting A*β*-induced microglial proliferation and impaired neurogenesis in mutant AD mice [56, 57]. Furthermore, altered signaling of GABA, which is essential for neuronal development, has been shown to depend on A*β* levels and to impact on differentiation and development of newborn neurons in the hippocampus of J20 mice [58]. Hence, on the basis of the above evidence, we suggest that A*β* accumulation is a key factor that triggers pathological events including altered neurotrophin levels, release of proinflammatory cytokines, and impaired GABA signaling, which in turn impact on AHN in AD brain.

#### 3.2.3. Apolipoprotein E (ApoE)

Apolipoprotein E (ApoE) is a molecule primarily secreted by astrocytes and involved in the regulation of lipid transport, synaptogenesis, and amyloid clearance [59, 60]. ApoE is also expressed by adult NSCs where it regulates their proliferative rate [61].

Among the allelic variants of ApoE gene, ApoE2 and ApoE3 are protective against the risk to develop AD, whereas ApoE4 associates with increased risk. Few studies investigated the contribution of ApoE variants to adult hippocampal neurogenesis, leading to mixed results.

As compared to relative controls, ApoE ko and ApoE4 mice display decreased dendritic complexity and spine density in adult-born neurons [62], indicating a reduced new-born neurons maturation.

Two studies [63, 64] reported that the expression of ApoE4 decreases adult neurogenesis in mutant mice as compared to wild type controls, while the expression of ApoE2 drives enhanced proliferation of DG progenitor cells and increased neurogenesis. Ageing in ApoE-expressing mice severely compromised neurogenesis, but the ApoE3 genotype was protective against this effect in female mice [64]. One other study [65] reported that the presence of ApoE4 was associated with enhanced neurogenesis in mice under standard conditions, whereas maintaining mice in environmentally enriched cages was associated with a drop of neurogenesis in ApoE4-expressing mice. Overall, these studies indicate that APOE polymorphisms play distinct roles in regulating the balance between neuronal birth and death.

#### 3.2.4. TAU

In physiological conditions, TAU phosphorylation facilitates migration of DCX^+^ cells [66], but levels of hyperphosphorylated TAU have been shown to be extremely high in neurogenic microenvironments of APP/PS1ΔΕ9 mice [67]. In particular, TAU immunoreactivity colocalized with BrdU^+^, GFAP^+^, and DCX^+^, meaning that alterations of TAU phosphorylation may be detrimental to NSCs, NPCs, and neuroblasts. One mechanism through which phosphorylated TAU can impact on AHN has been recently described in an important paper [68]. This study reported that phosphorylated TAU accumulates in GABAergic interneurons of the DG in both AD patients and 3xTg mouse model. The consequent GABA reduction, local circuit disinhibition and astrogliosis in turn impair AHN. The evidence that these alterations are reported in mice mutant for APP suggests a role for APP products in triggering TAU phosphorylation that alters AHN. Consistent with this view, we recently reported that, at least in the subventricular zone of an AD mouse model, NSCs fail to terminally differentiate due to TAU-mediated microtubule alteration, while blocking the generation of A*β* oligomers rescues this effect [69]. Hence, the induction of TAU phosphorylation can be one other mechanism through which APP products alter AHN in AD brains.

## 4. Rescue Neurogenesis in AD: Implications for Cognitive Recovery?

### 4.1. Specific Contribution of Altered Neurogenesis to Memory Decline in AD: Behavioral Studies in Animal Models

Among AD symptoms, proactive interference and cognitive rigidity are two key features that are in common with the effects of AHN alteration. In particular, persistence of old memories at the expense of new ones, a process known as anterograde amnesia, is a typical clinical sign of AD that also manifests in conditions of impaired neurogenesis. Despite anterograde amnesia in AD can be explained by neuronal damage that prevents the formation of new memories [70], no studies so far directly investigated AD-related anterograde amnesia in relation to AHN, leaving the door open to new explanations.

Instead, numerous studies in AD mouse models (see Table 2) have reported that altered neurogenesis associates with deficits in pattern integration, pattern separation, and cognitive flexibility. Pattern integration deficit in AD mouse models has been evidenced by impaired spontaneous alternation in the Y-maze task [57, 71–73], reduced step-down latency in the inhibitory avoidance test [74, 75], increased frequency to enter dark compartments of the passive avoidance apparatus [51], and reduced freezing in the fear conditioning task [43, 76]. In particular, impaired contextual but not cue fear conditioning [43] is indicative of a selective pattern integration deficit. Pattern separation deficit has been evidenced by low MWM performance in numerous studies [44, 46, 50–52, 57, 74, 75, 77–84] although most of these do not detail the experimental context which is essential to understand if the test is suitable for the evaluation of pattern separation effect. Other studies evidenced impaired pattern separation in AD mice by demonstrating that their reduced neurogenesis was associated with compromised performance in the object location task, which requires spatial discrimination [85], and in the object recognition task [73, 77]. Cognitive flexibility, the third function affected by impaired AHN, has been evidenced by one study reporting impaired reversal learning in the MWM task [80].

The overall picture emerging from the above studies is that impaired neurogenesis in AD mouse models is associated with deficits in one or more of the above-mentioned memory functions. Below, we review the most relevant studies that probed whether restoring neurogenesis correlates with memory recovery in these mice.

### 4.2. Factors That Rescue Neurogenesis and Recover AD Symptoms

#### 4.2.1. Enriched Environment and Physical Activity

**Figure 2:**
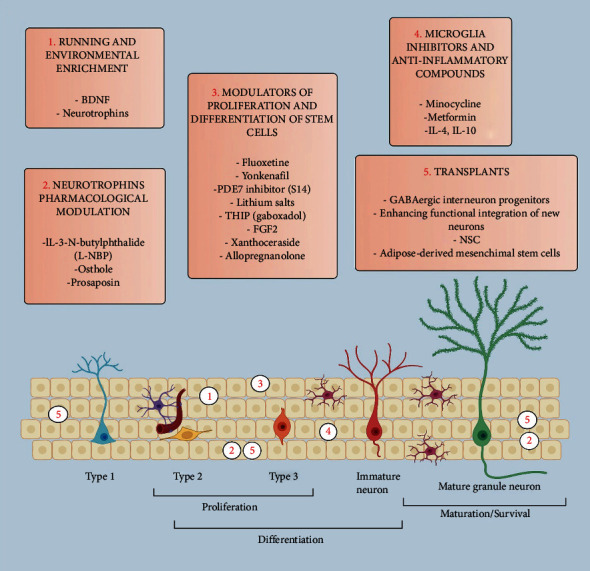
Factors that rescue AHN. Top boxes: factors that increase AHN. (1) Physical activity and environmental enrichment increase neurotrophin levels including BDNF, which in turn stimulate NSC proliferation and differentiation. Consistently, pharmacological modulation of neurotrophins (2) through compounds including L-NBP, Osthole, or Prosaposin can initiate NSC proliferation and also promote survival of newborn neurons. (3) Other modulators of proliferation and differentiation of stem cells can act to potentiate the generation of newborn neurons and their neuron fate specification. (4) Microglia can be found in 3 distinct states, each one affecting AHN through different mechanisms. Resting microglia ensure basal neurogenesis by releasing factors that control neuronal differentiation. Active microglia release proinflammatory cytokines that reduce NSC proliferation and differentiation. Congruent with this, microglia inhibitors and anti-inflammatory compounds rescue AHN in AD mice. Alternatively acting state microglia release anti-inflammatory cytokines including IL-4 and IL-10 that favor differentiation, viral-mediated expression of these cytokines reduces astro/microgliosis and enhances AHN. (5) Transplants of stem cells or progenitors as well as enhancing the functional integration of new neurons can potentiate AHN. Enhancing GABA signaling through transplant of GABAergic progenitors favors the maturation of newborn neurons. Transplanted NSCs in the hippocampus can differentiate in neurons or astrocytes. Mesenchymal stem cell transplantation increases neurogenesis by boosting differentiation and proliferation through mechanisms including the stimulation of alternatively activated microglia. Bottom panel: factors that increase AHN are represented along the stages of spatio-temporal transition of neural stem cell to a mature neuron. Numbers refer to the action of factors reported in top boxes. Please note that numbers position is indicative but not definitive as each factor can act through different mechanisms.

Since enriched environment and physical activity are potent neurogenic stimuli (Figure 2), numerous studies have investigated their potential therapeutic effect on AD mice. Although some of these studies found no evidence of a relation between environmental stimulation and AHN or improved memory [71, 86], other studies reported positive effects of environmental enrichment [87, 88] or its distinct components including social interaction and wheel-running.

Social enrichment, which increased BDNF protein and mRNA levels, was associated with improved AHN and FC memory in aged APP/PS1 mice [76], while social isolation worsened neurogenesis impairment in aged Tg2576 mice [43].

In one other study [89], environmental enrichment resulted in increased AHN, amelioration of MWM memory and upregulation of hippocampal neurotrophins in APP23 mice, yet these effects were not evident upon wheel-running only. However, one other study on APP/PS1 mice reported MWM rescue after wheel-running [78], thereby suggesting that this specific effect may be modulated by factors including genotype or overall running time.

In a pivotal study, Choi and collaborators [90] dissected the specific contribution of exercise-induced AHN in improving AD symptoms. They found that while genetically or pharmacologically induced AHN had little effect on AD symptoms in 5xFAD mice, running-induced AHN was associated with increased BDNF levels, reduced A*β* load, and improved cognition. Furthermore, combining genetically or pharmacologically induced AHN with BDNF administration was sufficient to rescue cognition, with no impact on A*β* levels. Overall, this study not only confirmed that physical activity can contrast AD symptoms but also demonstrated that BDNF significantly contributes to the therapeutic value of physical activity.

These animal studies are consistent with the reports of positive effects of physical activity in AD patients. In fact, epidemiological evidence indicates that physical activity as well as social and cognitive stimulation can delay dementia in aged individuals. Systematic and longitudinal studies in aged individuals with MCI have confirmed that physical activity can delay AD progression, reduce A*β* deposition and protect the brain from atrophy and temporal lobe volume loss [91, 92]. It has been documented that at least 12 months of mild-to-moderate physical activity (that is 50-70% of maximum cardiac output for 30-40 minutes session) can preserve cognitive function [93, 94].

However, it is yet to be demonstrated that these positive effects are directly mediated by activity-dependent increase of neurogenesis. Evidence rather suggests that the positive impact of physical activity on cognition may be unrelated to neurogenesis. One meta-analysis review indicated that the positive effects of physical activity on cognition are unlikely the results from changes in brain parameters [95]. Some studies evidenced that intense physical activity in humans was associated with an ameliorated pattern separation and mnemonic discrimination [96, 97]. However, these events were too close in time with physical activity to justify a possible involvement of neurogenesis. Our opinion is that physical activity in humans, by activating neurotrophins [98, 99] and by reducing the levels of inflammatory chemokines [100] could contribute to the rescue of both neurogenesis and memory in AD. Yet, it remains to be determined whether improved AHN can directly drive memory amelioration in AD patients.

#### 4.2.2. Treatments That Enhance AHN

A number of pharmacological approaches have been shown to effectively rescue AHN (Figure 2). Treatments that enhance neurotrophin levels can boost AHN by initiating NSC proliferation or by promoting survival of newborn neurons [101–103]. Among these treatments, the L-3-n-Butylphthalide (L-NPB) [52] or Osthole [51, 81] improved AHN and MWM performance in APP/PS1 mice. Similarly, administration of Prosaposin, a secreted protein that acts as trophic factor, rescued both loss of AHN and MWM impairment induced by intracranial A*β*1-42 injections in mice [45]. In a clinical trial performed in the 90s, neurotrophic factors were delivered by intracranial injection to the brains of a small group of AD patients. Despite modest cognitive improvement after short-term NGF infusion, this treatment was associated with severe side effects in the long term, making it not suitable for AD therapy [104–106].

Treatments that favor proliferation and differentiation of stem cells also ameliorate AHN and memory in AD mice. Fluoxetine, that increases progenitors' proliferation, restored memory in aged AD mice [43] while administration of phosphodiesterase inhibitors, which promotes cell differentiation, improved AHN and rescued object recognition, Y-maze and MWM in AD mouse models [57, 77]. Furthermore, lithium, which favors proliferation and neuron fate specification of newborn cells, was sufficient to rescue MWM in TgCRND8 mice [74].

Similarly, AAV-mediated expression of FGF2, a neurogenic factor for proliferation and differentiation, enhanced AHN and improved MWM memory in APP/PS1 mice [107]. One other relevant study used viral vectors to express a transcription factor necessary for maturation and survival of adult born cells. By enhancing maturation of granule cells in APP/PS1 mice, the authors found that amelioration in object location task was associated with a recovery of altered dendritic spines [85], indicating that newborn neurons that functionally integrate into hippocampal circuits are likely to support memory recovery. Microglia exert different functions on neurogenic processes, depending on their specific conformations [103]. As mentioned above, microglia at resting state contribute both to maintain RGLs in their quiescent state (qNSCs) and to release factors that promote neuronal differentiation in response to specific neurogenic stimuli [108]. Hence, microglia at this state are necessary to ensure basal neurogenesis. Conversely, active microglia inhibit NSC proliferation and differentiation through the release of proinflammatory cytokines [103]. Congruent with this, microglia inhibitors [109] and anti-inflammatory compounds like metformin [44] rescued both neurogenesis and memory in AD mice. A third state, the alternatively acting microglia, can release anti-inflammatory cytokines including IL-4 and IL-10 that favor differentiation [110, 111]. In line with this, viral-mediated expression of interleukin- (IL-) 4 or (IL-) 10 gene in the hippocampus of APP/PS1 mice resulted in enhanced AHN, reduced astro/microgliosis and A*β* deposition [112], and improved MWM learning [113]. Furthermore, administration of the nonsteroidal anti-inflammatory drugs (NSAIDs) such as indomethacin was associated with memory recovery in AD patients [114].

Transplant of NSCs that differentiate into neurons or astrocytes [115] has been proposed as therapeutic approach to contrast neuronal loss in AD. NSC transplant in the hippocampus APP/PS1 mice enhanced AHN as well as synaptic markers in hippocampal neurons. Interestingly, these changes were accompanied by amelioration in MWM and step-down test, suggesting that new neurons can integrate into synaptic circuits that sustain memory [75]. One other strategy consists in transplanting mesenchymal stem cells (MSCs) that can boost AHN through the expression of neurotrophins [116]. Transplant of adipose-derived MSCs in the hippocampus of APP/PS1 mice resulted in potentiated neurogenesis and novel object recognition ability [117]. As mentioned above, GABA signaling plays a key role in the maturation of adult-born neurons, and GABA imbalance has been found to link AD to impaired neurogenesis [118]. Restoring this signaling has potential therapeutic effects. Pharmacological strengthening of GABAergic function rescued AHN deficit with parallel amelioration of contextual memory in mice [68] and transplant of GABAergic interneuron progenitors in the DG of ApoE4 knock-in mice was sufficient to restore their MWM memory [119].

Hence, studies in AD mouse models have evidenced that treatments that boost AHN can also improve memory, but so far, the few treatments that have been formalized in clinical trials did not lead to the implementation of therapy for AD patients.

## 5. Conclusions

AHN loss has been reported in AD patients and mouse models before the clinical onset of disease [5, 120], in a preclinical stage commonly characterized by massive A*β* accumulation [121–123].

Here, we reviewed studies in AD animal models and we summarized the factors through which A*β* exerts its effects on AHN, including altered neurotrophins expression and the induction of proinflammatory cytokines. Targeting these factors by specific compounds can both ameliorate AD symptoms and rescue neurogenesis in AD mouse models; however, these results have not been translated into clinical trials.

Nevertheless, physical activity and a healthy lifestyle can restore neurotrophins and cytokine levels. Crucial questions therefore include which are the specific molecules that are modulated by physical activity in AD [124]. It is also of great relevance to investigate the concentrations or the temporal dynamics of such molecules, or what is their role in the amelioration of clinical symptomatology. In a wider perspective, focusing on those specific molecular targets can drive future clinical trials to set therapeutic strategies aimed at mitigating clinical symptoms in AD patients even in conditions that preclude their physical activity.

Despite a significant progress in the study of AD and AHN over the past decades, it still remains to be determined whether AHN loss has a causal role in cognitive decline in AD. Studies in Table 2 sustain this possibility in mouse models, but this scenario cannot be extended to AD patients due to the impossibility to perform similar experimental studies in humans. Furthermore, comparisons between rodents and humans are limited by the fact that the rate of AHN is much higher in rodents than in humans.

Studies in humans should rather demonstrate that pharmacological interventions that clearly restore AHN also delay or contrast cognitive decline. To this aim, researchers should combine innovative clinical trials, like mesenchymal stem cell transplantation [125], with precise imaging tools that may allow for AHN identification and with specific behavioral studies investigating pattern integration, pattern separation, and memory erasure, the key memory functions associated with AHN.

## Figures and Tables

**Table 1:** Studies investigating AHN on AD mouse models.

Mouse model	Age	BrdU treatment	BrdU	DCX	BrdU/DCX	OTHER markers	Ref
APP/PS1	8 months	4 days - analysis 6 days later	↓	↓		↓ dendritic branches, ↓ dendritic spines, ↓synaptic markers	46
	10 months	-		↓		↓ dendritic branches, ↓synaptic markers	47
	8-9 months	7 days - analysis 18 hours later	↓			= Nestin, = Ki67/Nestin	51
	6 months	4 days (twice per day) - analysis 24 hours later	↓				52
	2, 4, 6 months	3 days - analysis 3 days later		↓ at 4 and 6 months	↓ at 6 months	↓ BLBP+ stem cells at 6 months	56
	12 months	3 days (twice per day) - analysis 2 weeks later	↓	↓		↓ Nestin, ↓ synaptic proteins	75
	12 months	7 days - analysis 1 day later	↓				78
	8 months	-				↓ Sox2	83
	2 months	EdU 7 days - analysis one month later				↓ EdU, ↓ EdU/NeuN	87
	3 months	3 days (twice per day) - analysis 5 weeks later	↓BrdU/NeuN			↓dendritic ramification ↓Spine density	109
	8 months	3 weeks - analysis at the end	↓	↓		↓BrdU/NeuN	113
	7 months	12 days - analysis 1 day (a) or 30 (b) days later	= (a), ↓ (b)		↓ (b)	= Ki67+ cells, ↓ Brdu+/NeuN+/S100b-,↓ Brdu+/DCX+/NeuN-, ↓ Brdu+/DCX-/NeuN+	127

APP/PS1ΔΕ9	28 weeks	7 days (twice per day) - analysis 7 days later	↓	↓		↓ NeuN	44
	3 months	3 days - analysis 2 hours (a) or 4 weeks (b) later	=	↓		↓ NeuN	57
	2 months	3 days (twice per day)	↓		↓		67
	3,9,18 months	1 day - analysis 18 hours later	= (all ages)	↓ at 18 months		↓ total DG cells at 18 months	72
	3 months	3 days - analysis 1 day later	↓	↓		↓ NeuN	73
	4, 10 months	2 days (a), 3 days (b) - analysis 1 day later (a), (b)	↓ at 4 and 10 months	↑ at 3 months, = at 10 months			80
	9 months	-				↓Ki67, ↓synaptic proteins	82
	8-9 months	-				↓ newborn (GFP+), ↓ spine density	85
	2 months	1 injection - analysis 1 day and 14 days later	↓ in enriched mice		↓ in enriched mice	↓ DG cells and ↓ BrdU/NeuN in enriched mice	86
	3 months	3 days - analysis 3 hours (a) and 4 weeks later (b)	↓(a), = (b)		↓(a), = (b)	= (b) BrdU/NeuN	88
	3,5,10,15months	1 injection - analysis 1 day later	↓ at 3, 5, 10 months	↓			126
	3,10,13 months	5 days - analysis 4 weeks later	↓	=	=	↑ PCNA, ↑ PCNA/DCX at 3 months, ↓BrdU/NeuN	128
	9,13 months	4 days (twice per day) - analysis at day 4	↓			↓ GFAP/Sox	129

APP/PS1/Nestin-GFP	7 days; 1, 3 7 months	1 day - analysis 2 (a) or 3(b) hours later	↓			↓ Nestin, ↑ DCX/Nestin at 3 and 7 months, ↓ Spine density at 3 and 7 months	84

PS1M146L	2 months	1 injection - analysis 1 day and 14 days later	↓ in enriched mice		↓ in enriched mice	↓ BrdU/Tub and BrdU/NeuN in enriched mice	86

PS1/PS2-KO	7-9, 18-20 months	1 injection - analysis 1 day later	↑	↑		↑Brdu/NeuN, ↑ BrdU/GFAP	130

APP/PS1 KI	2, 6 months	-		↓ at 6 months			71
	6 months	-		↓			79

aAPP751	1,3,6,9 months	1 day before grafting - analysis 1,3,6,9 months later	↓	↓		↓ Synaptic markers	55

APPSw,Ind	4 months (plus 7 weeks EE)	5 days- analysis 7 weeks later	↓	↓		↓BrdU/NeuN, ↓ dendritic ramification, ↓ DG volume	50

Tg2576	3,6, 9 months	3 days - analysis 1 day later	↓				43
	3,5 months	-		=		↑ PCNA, ↑ PCNA/DCX at 3 months	128
	3, 5, 12, months	1 day - analysis 1 day (a) or 30 days (b) later	↓ at 3 months			↓ Brdu/NeuN, ↓ dendritic ramification, ↓ spine density at 3 months	131
						BrdU+/DCX+/NeuN+ at 3 months, Brdu+/DCX-/NeuN- at 12 months	

Tg19959	5 weeks; 10, 15 months	-		↑ at 5 weeks		↑ Ki67	132

Arg-61	3-12 months	-		↑ at 3 months, ↓ at 12 months			133

3xTgAD	3 months	1 day - analysis 1 day later	↓				48

PDAPP	2, 12 months	1 injection - analysis 2 hours (a) and 4 weeks (b) later	↓(a) and (b)	↓(a)		= Brdu/NeuN and =BrdU/GFAP (b)	134

PDGF-APP	3,12 months	3 days - analysis 7 days later	↑	↑			34

CRND8	3, 7 months	3 days - analysis 1 day (a) and 5 weeks (b) later	↓(a) and (b)	↓(a)		↓BrdU/NeuN (b)	74
	6, 7, 9, 11, 13 weeks	5 days - analysis 1 (a), 2 (b), 4 (c), 6 (d), 8 (e) weeks later	↑			= BrdU/NeuN at 1 and 8 weeks; ↓ Brdu/GFAP (a), = (e)	135

ApoE3, ApoE4	10 weeks	3 days (twice per day) - analysis 4 weeks later	=			= BrdU/Prox1, = Ki67/GFP/Nestin , ↓ dendritic ramification and ↓ spine density in ApoE4 vs ApoE3	62

ApoE3, ApoE4	6-7 months	1 day - analysis 1 day (a), 3 days (b), 4 weeks (c) and 10 weeks (d) later	↑ ApoE4 (a,c)		↑ ApoE4 (c)	↓ Brdu/Neun (c,d), ↑ ki67 (a), ↓ dendritic branching and ↓ GAD67 in ApoE4	63

ApoE2, ApoE3, ApoE4	10-12 weeks; 1 year	3 days - analysis 1 day later	↓ ApoE4, ↑ ApoE2/3, age- and sex-dependent				64

ApoE4	9-15 months	-	=				136

ApoE ko	10 weeks	3 days (twice per day) - analysis 4 weeks later	=			= BrdU/Prox1, ↓Ki67/GFP/Nestin, ↓ dendritic ramification , ↓ spine density	62
	6-7 months	1 day - analysis 1 day (a), 3 days (b), 4 weeks (c) and 10 weeks (d) later	↓ (b)		↓(c,d)	↓ Brdu/Neun (c,d) , ↑ BrdU/S100b (b,c,d)	63
	9-15 months	-		↑ in female, = in male			136

Table summarizes the studies investigating neurogenesis in distinct AD mouse models (column 1) at different age points (column 2). Column 3 reports details of BrdU treatment (days/number of injections and time between last BrdU injection and animal sacrifice). Columns 4, 5, and 6 refer, respectively, to quantifications of BrdU^+^, DCX^+^, and BrdU^+^/DCX^+^ cells in the dentate gyrus (DG) region. Column 7 refers to other neurogenic/synaptic markers found in the DG. Symbols indicate increase (↑), decrease (↓) or no variation (=) of specific markers as compared to wild type controls. Details including different time points (a, b) between BrdU and sacrifice of the animal are reported where necessary. Unless otherwise indicated, male mice were used in the studies. BrdU: bromodeoxyuridine; DCX: doublecortin; Ki67: proliferative marker; BLBP: brain lipid-binding protein; Nestin: nestin protein; Sox2: SRY- (sex determining region Y-) box; EdU: ethynyldeoxyuridine (BrdU analogue); S100B = S100 calcium-binding protein B; PCNA: proliferating cell nuclear antigen; GFAP: glial fibrillary acidic protein; Tub: tubuline; GAD67: glutamic acid decarboxylase 67 (GABA-synthesizing enzyme).

**Table 2:** Studies investigating memory in relation to AHN in AD mouse models.

Mouse model	Age	Test	Behavioural outcome	Increased neurogenesis by	Ref
APP/PS1	8 months	MWM	↑ escape latency, ↓ time in target quadrant, ↓ platform crossings	b2AR activation (clenbuterol)	46
	8-9 months	PA (a)	↓ latency and ↑ frequency to enter in dark compartment	osthole (a), (b)	51
		MWM (b)	↑ escape latency, ↑ distance from platform, ↓ platform crossings		
	6 months	MWM	↑ escape latency	L-3-n-butylphthalide (L-NBP)	52
	12 months	MWM (a)	↑ escape latency, ↓platform area crossings, ↓ time spent in target quadrant	NSC translpant (a), (b)	75
		SD (b)	↓ latencies; ↑ error time		
	1,3,6,9,12 months	FC	↓ freezing from 6 months of age	social interaction	76
	2 months	EPM (a)	= time and number of entries in open arms	enriched environment (a)	87
		FC (b)	= time in freezing	-	
	6 months	NOR (a)	↓ recognition index	PDE7 inhibitor (S14) (a), (b)	77
		MWM (b)	↑ escape latency		
	12 months	MWM	↑ escape latency, ↓ time in target quadrant, ↓ platform area crossings	running	78
	9 months	MWM	↑ escape latency, ↓ platform area crossings, ↓ time in target quadrant	osthole	81
	8 months	MWM	↑ escape latency, ↓ time in target quadrant	MDA7 (CB2 receptors agonist)	83
	4, 7-8 months	MWM (radial version)	↑ error rate	FGF2	107
	18 weeks	NOR (a)	↓ recognition index	minocycline (a)	109
		YM (b)	= alternation rate		
	7 months	MWM (radial version)	↑ error rate	IL-10	113

APP/PS1ΔΕ9	28 weeks (female)	MWM	↑ escape latency, ↓ time in platform area, ↓ platform crossings	metformin	44
	3 months	nest building behavior (a)	↓ nesting score	yonkenafil (a, dose-dependent), (b), (c, dose-dependent)	57
		SA (b)	↓ alternation and arm entries		
		MWM (c)	↓ escape latency, ↓ platform crossings		
	9-18 months	SA	↓ alternation rate at 18 months age	Paroxetine	72
	3 months	OF (a)	= locomotor activity	xanthoceraside (b, dose-dependent), (c, dose-dependent)	73
		YM (b)	↓ spontaneous alternation		
		NOR (c)	↓ discrimination index		
	3, 9 months	NOR	= exploration for novel object at 3 months	-	80
		Locomotor activity	↑ activity at 3 months	-	
		YM	= time in open arms at 3 months	-	
		MWM	↑ escape latency at 9 months	-	
	9 months	MWM	↑ escape latency, ↓ platform area crossings and time in target quadrant	osthole	82
	8-9 months (female)	OL	↓ exploratory preference for displaced object	enhancing functional integration of new neurons	85

APP/PS1 KI	6 months	EPM (a)	↑ time in open arms	-	71
		YM (b)	↓ alternation rate	-	
	2-6 months	neurological evaluation (a)	↓ of vertical activity (age-related), = other parameters, hyperactivity	-	79
	6 months	EPM (b)	↑ time in open arms	-	
	2, 4, 6 months	MWM (c)	↓ in goal target (age-related), ↓ accuracy (age-related)	-	

APPSw,Ind	4 months (plus 7 weeks EE)	MWM	↑ escape latency, ↓ time in platform area, ↓platform crossings	Environmental Enrichment	50

Tg2576	3,6, 9 months	FC (contextual and cue)	↓ freezing to context after stress (isolation) at 6 and 9 months	Fluoxetine	43

3xTgAD	6 months	OL	↓ exploratory preference for displaced object	THIP (Gaboxadol)	68
		FC (pattern separation)	↑ trials for discrimination		
	3 months	TEC	↓ conditioned responses	Allopregnanolone	48

CRND8	3, 7 months	SD (a)	↓step-down latency	Lithium salts (a) 3 months only, (b) 3 months only	74
		MWM (b)	↑ escape latency, ↓ time in target quadrant		

ApoE3-Ki and ApoE-4 KI	10-17 months	MWM (a)	↓ ApoE4-KI preference for target quadrant (respect to Apoe3-KI)	Transplant of embryonic interneuron progenitor (a), (b), (c)	119
		OF (b)	↓ ApoE4-KI time in central area (respect to Apoe3-KI)		
		EPM (c)	↓ ApoE4-KI time in open arms (respect to Apoe3-KI)		

Table summarizes studies investigating neurogenesis and memory in distinct AD mouse models (column 1) at different age points (column 2). Column 3 reports the memory test(s) used in each study. Column 4 refers to results from each memory test with symbols indicating increase (↑), decrease (↓), or no variation (=) of specific behavioral outcomes as compared to wild type controls (unless otherwise indicated). Please note (↑) latency corresponds to lower memory. When available, treatments that were associated with a rescue in neurogenesis and also ameliorated memory are reported in column 5. MWM: Morris water maze (standard or radial); PA: passive avoidance; SD: step down; FC: fear conditioning (contextual or cue); EPM: elevated plus maze; NOR: novel object recognition; OF: open field; YM: Y maze; SA: spontaneous alternation; OL: object location; TEC: trace eye-blink conditioning.
